# A single-dose, randomized, open-label, four-period, crossover equivalence trial comparing the clinical similarity of the proposed biosimilar rupatadine fumarate to reference Wystamm^®^ in healthy Chinese subjects

**DOI:** 10.3389/fphar.2024.1328142

**Published:** 2024-05-17

**Authors:** Sisi Lin, Yutao Lou, Rui Hao, Yiming Shao, Jin Yu, Lu Fang, Meihua Bao, Wu Yi, Yiwen Zhang

**Affiliations:** ^1^ Department of Pharmacy, Zhejiang Provincial People’s Hospital, People’s Hospital of Hangzhou Medical College, Hangzhou, China; ^2^ Hunan Key Laboratory of the Research and Development of Novel Pharmaceutical Preparations, School of Pharmaceutical Science, Changsha Medical University, Changsha, China; ^3^ Key Laboratory of Endocrine Gland Diseases of Zhejiang Province, Hangzhou, China; ^4^ Zhejiang Provincial Clinical Research Center for Malignant Tumor, Hangzhou, China

**Keywords:** rupatadine fumarate, pharmacokinetics, bioequivalence, reference-scaled average bioequivalence, safety

## Abstract

**Purpose:**

The aim of this study was to evaluate the bioequivalence of two formulations of rupatadine (10-mg tablets) under fasting and fed conditions in healthy Chinese subjects.

**Methods:**

A total of 72 subjects were randomly assigned to the fasting cohort (*n* = 36) and fed cohort (*n* = 36). Each cohort includes four single-dose observation periods and 7-day washout intervals. Blood samples were collected at several timepoints for up to 72 h post-dose. The plasma concentration of rupatadine and the major active metabolites (desloratadine and 3-hydroxydesloratadine) were analyzed by a validated HPLC–MS/MS method. The non-compartmental analysis method was employed to determine the pharmacokinetic parameters. Based on the within-subject standard deviation of the reference formulation, a reference-scaled average bioequivalence or average bioequivalence method was used to evaluate the bioequivalence of the two formulations.

**Results:**

For the fasting status, the reference-scaled average bioequivalence method was used to evaluate the bioequivalence of the maximum observed rupatadine concentration (C_max_; subject standard deviation > 0.294), while the average bioequivalence method was used to evaluate the bioequivalence of the area under the rupatadine concentration–time curve from time 0 to the last detectable concentration (AUC_0-t_) and from time 0 to infinity (AUC_0-∞_). The geometric mean ratio (GMR) of the test/reference for C_max_ was 95.91%, and the upper bound of the 95% confidence interval was 95.91%. For AUC_0-t_ and AUC_0-∞_ comparisons, the GMR and 90% confidence interval (CI) were 98.76% (93.88%–103.90%) and 98.71% (93.93%–103.75%), respectively. For the fed status, the subject standard deviation values of C_max_, AUC_0-t_, and AUC_0-∞_ were all <0.294; therefore, the average bioequivalence method was used. The GMR and 90% CI for C_max_, AUC_0-t_, and AUC_0-∞_ were 101.19% (91.64%–111.74%), 98.80% (94.47%–103.33%), and 98.63% (94.42%–103.03%), respectively. The two-sided 90% CI of the GMR for primary pharmacokinetic endpoints of desloratadine and 3-hydroxydesloratadine was also within 80%–125% for each cohort. These results met the bioequivalence criteria for highly variable drugs. All adverse events (AEs) were mild and transient.

**Conclusion:**

The test drug rupatadine fumarate showed a similar safety profile to the reference drug Wystamm^®^ (J. Uriach y Compañía, S.A., Spain), and its pharmacokinetic bioequivalence was confirmed in healthy Chinese subjects based on fasting and postprandial status.

**Clinical trial registration:**

http://www.chinadrugtrials.org.cn/index.html, identifier CTR20213217

## 1 Introduction

Allergic rhinitis is a common clinical condition in otorhinolaryngology. Epidemiological studies showed that the global incidence of the disease is 10%–40% in adults and 2%–25% in children (Asher et al., 2006; Hoyte and Nelson, 2018). In China, the incidence of allergic rhinitis fluctuates between 8% and 25% in adults and between 10% and 22% in children (Zhang et al., 2009; Wang et al., 2016; Pang et al., 2022). Urticaria is also a common skin condition characterized by wind clumps, angioedema, or both (Zuberbier et al., 2014). It manifests as acute and chronic urticaria, affecting approximately 20% and 5% of the general population, respectively; moreover, these rates are increasing annually (Fine and Bernstein, 2016; Antia et al., 2018; Maurer, Zuberbier, and Metz, 2022). In addition to elevating medical expenses, allergic rhinitis and urticaria impose a heavy economic burden on the society; they also exert a detrimental effect on the ability of patients to work, sleep, interact socially, and even manage their emotions. These effects have a significant negative impact on quality of life and are associated with a significant healthcare burden. Therefore, the control of the disease progression is clinically significant for improving the quality of life, daily activities, and wellbeing of patients.

Rupatadine, a second-generation H1 antihistamine, was initially approved for the treatment of allergic rhinitis and urticaria by the European Medicines Agency in 2002 (Valero et al., 2009). Rupatadine exhibits anti-allergic and anti-inflammatory characteristics, with good safety features. This is shown by the inhibition of mast cell degranulation due to immunological or non-immune system stimulation and inhibition of mediators involved in the inflammatory response (Mullol et al., 2008). This agent has demonstrated clinical benefits in relieving symptoms of nasal airway, rhinocnesmus, and obstruction due to a runny nose (Picado, 2006; Fantin et al., 2008; Mullol et al., 2019; Muñoz-Cano et al., 2019). Moreover, it alleviates clinical symptoms in patients with urticaria (Hide, et al., 2019a). As a prodrug, rupatadine has a rapid onset of action and is extensively metabolized by P450 3A4, yielding desloratadine and 3-hydroxydesloratadine as the major active metabolites (Valero et al., 2009). The within-subject variabilities for rupatadine in the maximum observed rupatadine concentration (C_max_) (coefficient of variation [CV]: −38.8%) and area under curve (AUC; CV: −33.9%) are considerable (Merlos et al., 1997; Picado, 2006). According to the U.S. Food and Drug Administration and the National Medical Products Administration of China (NMPA) guidelines on bioequivalence studies, the reference-scaled average bioequivalence (RSABE) approach is recommended for evaluating the bioequivalence of highly variable drugs (U.S. Food and Drug Administration, 2001; The NMPA, Center for Drug Evaluation, 2019).

Rupatadine fumarate tablet (10-mg tablets, T) is a generic drug, developed by Haisco Pharmaceutical Group Co., Ltd. (Meishan, China) and launched in China in 2014. Its active ingredient, dosage form, specifications, indications, route of administration, and dosage are consistent with those of the reference drug Wystamm^®^ (10-mg tablets, R). The generic drugs have lower costs than original products, providing a potential method to overcome the economic burden on patients. Bioequivalence (BE) studies comparing generic to innovator products are required for marketing a new generic product by the NMPA of China. A systemically active generic drug is considered to be bioequivalent to the reference drug if the rate and extent of absorption of the two products do not show any significant difference, which is assessed by conducting BE studies in human subjects to compare their pharmacokinetic characteristics. The purpose of our study was to 1) evaluate the safety and PK parameters of rupatadine and its active metabolites and 2) compare the bioequivalence of two rupatadine fumarate (10-mg tablets) formulations acquired from different suppliers.

## 2 Methods

### 2.1 Subjects

The study was performed at the Zhejiang Provincial People’s Hospital-Phase I Clinical Research Center (Hangzhou, China) from December 2021 to March 2022. All subjects were informed regarding the study and provided written informed consent prior to their participation. The inclusion criteria are as follows: healthy male and female subjects aged 18–45 years; weight ≥50 kg for male subjects and ≥45 kg for female subjects; body mass index 19.0–26.0 kg/m^2^; and full understanding of the informed consent, test content, process, and possible adverse events (AEs).

The exclusion criteria included the following: a history/presence of any clinically relevant condition or disease; clinically significant abnormal physical examination, electrocardiogram, laboratory, or viral serology tests; HBV Ag, HCV Ab, HIV Ab, and TP Ab tests have clinical significance; have special dietary requirements or a history of dysphagia or lactose intolerance; allergic constitution or known allergy to the study drug; cannot tolerate venipuncture or a history of needle sickness and blood sickness; have had a special diet or strenuous exercise within 2 weeks prior screening, which will affect the drug ADME prediction; a history/presence of alcohol and/or smoking and/or drug abuse; treatment with a drug that affects liver drug enzymes within 4 weeks or other drugs within 1 week prior to enrollment, received live vaccination within 3 months or plan to be vaccinated during the trial; a history of participation in other drug clinical trials, or blood loss > 450 mL within 3 months; and subjects deemed unsuitable for participation by investigators. Pregnant or lactating women were also excluded.

### 2.2 Study design

The protocol, amendments, and informed consent forms were approved by the Ethics Committee of the Zhejiang Provincial People’s Hospital. The study was conducted in accordance with the Declaration of Helsinki (World Medical Association, 2013), principle of Good Clinical Practice (NMPA, National Health Commission of the People’s Republic of China, 2020), and Chinese laws and regulations.

**FIGURE 1:**
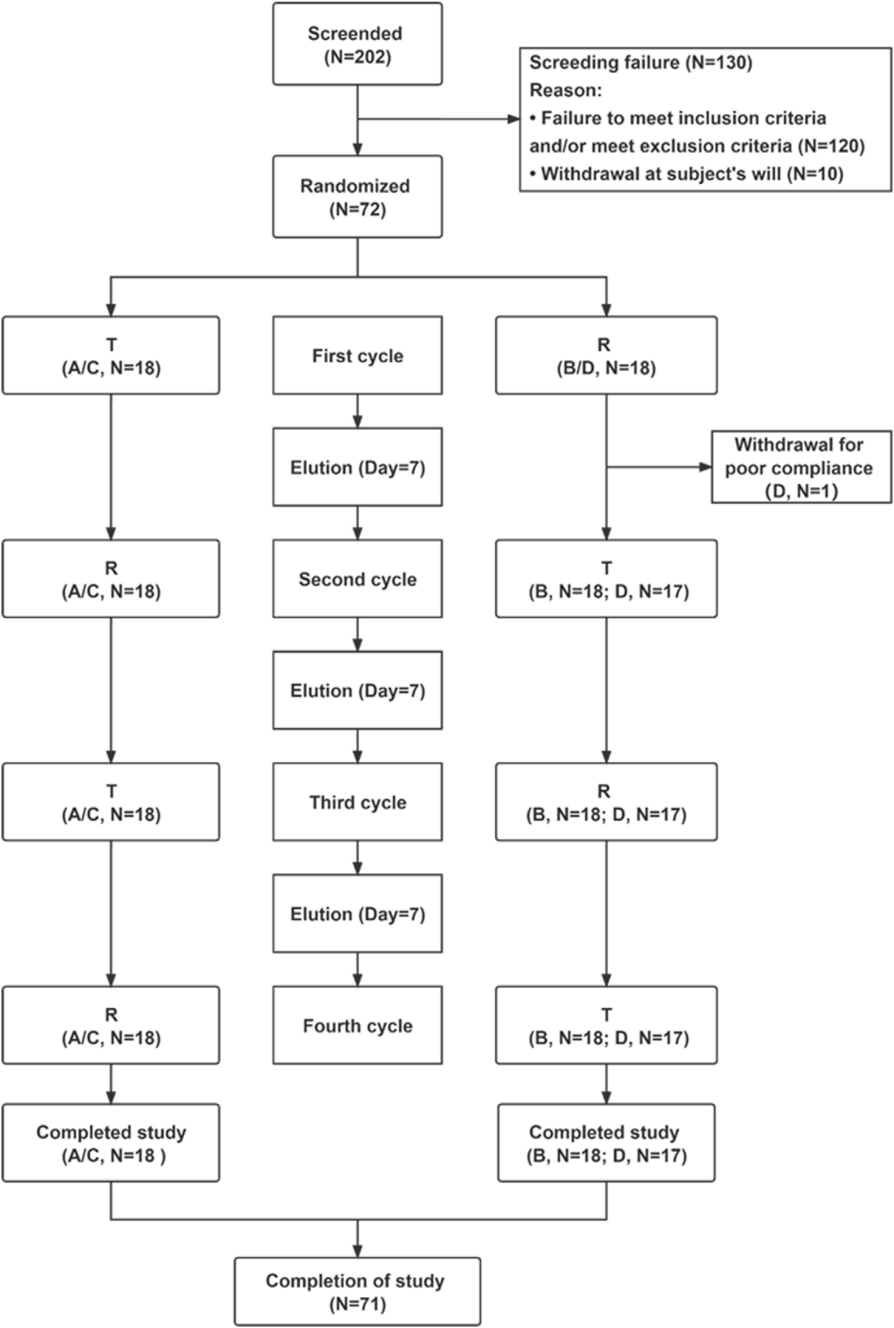
Flow chart of the study. Abbreviations: T, test; R, rupatadine; N, number of subjects. Notes: reference indicates Wystamm^®^; A and B represent groups of the fasting status; and C and D represent groups of the postprandial status.

According to the NMPA guidelines (NMPA, Center for Drug Evaluation, 2019), this study was an open-label, randomized, two-treatment, full-replicated, four-period, two-sequence crossover design that was conducted in two cohorts under fasting and fed conditions. The trial featured a screening period, four treatment periods, a washout period of 7 days after each treatment period, and a follow-up period following the last treatment (Figure 1). Healthy subjects were randomly assigned to sequences of RTRT or TRTR by a randomized block design using SAS version 9.4 software. In each treatment period, all subjects received a single oral administration of 10 mg of rupatadine fumarate (specification: 10 mg; lot number: 210703; Haisco Pharmaceutical Co., Ltd.) or Wystamm^®^ (specification: 10 mg, lot number: P003; J. Uriach y Compañía, S.A.). In the fasting cohort, subjects received rupatadine following an overnight fasting period (≥10 h). After a 7-day washout period, the subjects received orally the same dose of another formulation of rupatadine following a sequence of RTRT or TRTR. In the fed cohort, subjects consumed a high-fat breakfast (containing 528.3 calories of fat, 274.8 calories of carbohydrate, and 180.4 calories of protein) within 30 min before dosing. Investigational drugs were administered using 240 mL of water under supervision by a qualified pharmacist. Subjects were not allowed to drink additional water for 1 h before and after treatment. Food intake was strictly controlled, and standardized lunch and dinner were provided approximately 4 and 10 h post-administration, respectively.

### 2.3 Sample collection and analysis

Blood samples (4 mL) were collected before treatment (≤30 min) at 10 min, 20 min, 30 min, and 45 min and at 1, 1.25, 1.5, 2, 2.5, 3, 3.5, 4, 5, 6, 8, 12, 24, 48, and 72 h post-dose in K_2_-ethylenediaminetetraacetic acid anticoagulation tubes by direct venipuncture. After sample collection, plasma was separated by centrifugation (1,700 *g* × 10 min, 4°C) and stored in a −70°C freezer for 2 h until transfer to the analysis department. The plasma concentrations of rupatadine, desloratadine, and 3-hydroxydesloratadine were analyzed using a validated liquid chromatography–tandem mass spectrometry method (Sun et al., 2015). The quantitative range of the standard curve was 0.05–10.0 ng/mL for rupatadine and 0.025–5.00 ng/mL for desloratadine and 3-hydroxydesloratadine.

### 2.4 Pharmacokinetic analysis

This study evaluated the bioequivalence of rupatadine fumarate, a generic chemical drug of Wystamm^®^, using PK parameters as endpoints. The main PK parameters were C_max_ and area under the rupatadine concentration–time curve from time 0 to the last detectable concentration (AUC_0-t_) and from time 0 to infinity (AUC_0-∞_). Secondary PK parameters included the time of the maximum measured plasma concentration (T_max_), elimination half-life (t_1/2_), and terminal rate constant (λ_z_). Both the main and secondary PK parameters were calculated using the non-compartmental analysis model with Phoenix^®^ WinNonlin 8.3 (Certara, Princeton, New Jersey).

### 2.5 Safety evaluation

Safety was evaluated at the screening period, treatment period, and follow-up period. The evaluation included AE monitoring throughout the study period, physical examination, vital signs (i.e., temperature, blood pressure, and pulse rate), clinical laboratory tests (i.e., blood routine, urine routine, blood biochemistry, and coagulation), 12-lead electrocardiogram, and pregnancy screening (female subjects only). All AEs were recorded immediately by the clinical research physician, and the relationship with the study drug and severity were evaluated with reference to the Common Terminology Criteria for the Evaluation of Adverse Events (version 5.0). In the safety analysis set, AEs were coded using the Preferred Terminology of the International Medical Terminology Dictionary (MedDRA version 24.1) and summarized according to the systematic organ classification.

### 2.6 Sample size and statistical analysis

According to a previous study (Solans et al., 2007), the total CV for C_max_ was 56.9% and 59.8%, respectively, and the total CV for AUC_0-t_ was 62.9% and 50.8%, after a single administration of rupatadine tablets under fasting and fed conditions, respectively. Assuming no influence of food intake on the PK parameters of rupatadine, intra-individual CV for C_max_ and AUC_0-t_ was estimated to be 38.8% and 33.9%, respectively, based on a 90% confidence interval (CI) for fasting *versus* fed conditions. Therefore, the intra-individual CV of AUC_0-t_, AUC_0-∞_, and C_max_ for the reference drug Wystamm^®^ in this study was overestimated to be 38%. Assuming that the geometric mean ratio (GMR) of the two formulations ranges from 0.95 to 1.05, with a one-sided test type I error probability α = 0.05 (two-sided total of 0.10), it was estimated that a minimum of 34 subjects would be required for the equivalence test method of the four-cycle crossover trial. This sample size would guarantee that the 90% CI for the GMR of the main PK parameters (AUC_0-t_, AUC_0-∞_, and C_max_) for the test and reference drugs with >80% power was between 80.0% and 125.0%. Considering the possibility of dropout, it was planned to recruit 36 subjects (18 subjects per administration group). Therefore, it was decided that the final sample size for the fasting status and postprandial status study would be 72 subjects.

Following the natural logarithmic transformation of the main PK parameters (C_max_, AUC_0-t_, and AUC_0-∞_), the fixed-effect analysis of variance (ANOVA) model was used to analyze statistical significance. Statistical analysis was performed using a two-sided test and 90% Cl to evaluate the bioequivalence between the test drug rupatadine fumarate and the reference drug Wystamm^®^. Prior to the evaluation of bioequivalence, the within-subject CV (CV_WR_) was calculated for each PK parameter of Wystamm^®^. If the CV_WR_ was <30% for the main PK parameters (i.e., C_max_, AUC_0-t_, and AUC_0-∞_), the PK bioequivalence evaluation was conducted using the average bioequivalence (ABE) method (U.S. Food and Drug Administration, 2001). A 90% CI of the GMR for the main PK parameters between the predefined intervals of 80.0%–125.0% denoted PK bioequivalence for the relevant reference product. If CV_WR_ was ≥30% for the main PK parameters, the RSABE method was performed for bioequivalence evaluation (Davit et al., 2012). In this case, PK bioequivalence was concluded if the upper limit of the one-sided 95% CI for ${\left(Y_{T}-Y_{R}\right)}^{2}-\theta S_{\text{WR}}^{2}$ (calculated based on Howe’s approximation Ⅰ) was ≤0 and the GMR of the main PK parameters was between 80.0% and 125.0%. The Wilcoxon rank-sum test was used to assess T_max_ and t_1/2_, and t-tests were used to compare other PK parameters between the two groups. All statistical analyses were carried out using SAS version 9.4 software (SAS Institute Inc., Cary, NC, United States).

## 3 Results

### 3.1 Subject demographics and baseline characteristics

Of the 202 subjects initially screened in this study, 130 did not meet the inclusion criteria and were excluded (Figure 1). The remaining 72 subjects were randomly divided into four groups (18 subjects per group): group A or group B (fasting status) and group C or group D (postprandial status). Only one subject in group D dropped out of the study due to poor adherence, while all other subjects completed the study. Demographic features and baseline clinical characteristics (listed in Table 1) were comparable between the treatment groups. There were no significant differences in the demographic characteristics between the two sequences for the fasting and fed conditions.

**TABLE 1:** Subject demographics and baseline characteristics by treatment sequence.

	Fasting status			Fed status		
	Group A	Group B	*p*-value (*t*-test)	Group C	Group D	*p*-value (*t*-test)
	(N = 18)	(N = 18)		(N = 18)	(N = 17)	
Age, years						
Mean ± SD	28.8 ± 7.13	28.1 ± 7.27	0.7723	28.2 ± 5.01	28.6 ± 5.96	0.8308
Median (Q1 and Q3)	27.0 (23.0, 32.0)	25.0 (23.0, 33.0)		28.5 (24.0, 32.0)	29.5 (24.0, 32.0)	
Min–max	20, 43	21, 44		20, 38	18, 42	
Sex, N, %						
Male	14 (77.8)	14 (77.8)		15 (83.3)	16 (88.9)	
Female	4 (22.2)	4 (22.2)		3 (16.7)	2 (11.1)	
Ethnicity, N, %						
Han	16 (88.9)	17 (94.4)		15 (83.3)	17 (94.4)	
Others	2 (11.1)	1 (5.6)		3 (16.7)	1 (5.6)	
Height, cm						
Mean ± SD	166.31 ± 4.42	170.64 ± 5.70	0.0156	169.39 ± 5.74	170.92 ± 6.28	0.4568
Median (Q1 and Q3)	167.00 (161.50, 170.00)	170.00 (167.00, 173.50)		170.00 (164.50, 173.00)	169.50 (165.50, 177.50)	
Min–max	155.0, 175.5	161.5, 185.5		160.5, 181.5	162.5,182.5	
Weight, kg						
Mean ± SD	63.47 ± 6.09	66.28 ± 8.57	0.2648	65.48 ± 7.76	68.34 ± 7.51	0.2763
Median (Q1 and Q3)	62.85 (60.00, 67.30)	65.5		64.65 (59.50, 71.00)	67.80 (62.30, 75.40)	
		(61.80, 70.20)				
Min–max	51.6, 74.7	52.7, 87.0		52.6,76.7	58.1, 82.6	
BMI, kg/m2						
Mean ± SD	22.94 ± 1.53	22.68 ± 1.80	0.6435	22.75 ± 1.70	23.33 ± 1.51	0.2947
Median (Q1 and Q3)	23.15 (22.20, 23.70)	22.95 (21.30, 24.00)		23.20 (20.80, 24.00)	23.20 (22.10, 25.10)	
Min–max	20.4, 25.9	19.4, 25.6		19.8, 25.9	20.8, 25.6	
Current smoking, n (%)	0 (0.0)					
Combined drug and non-drug treatment status, n (%)	0 (0.0)					

### 3.2 PK properties

**FIGURE 2:**
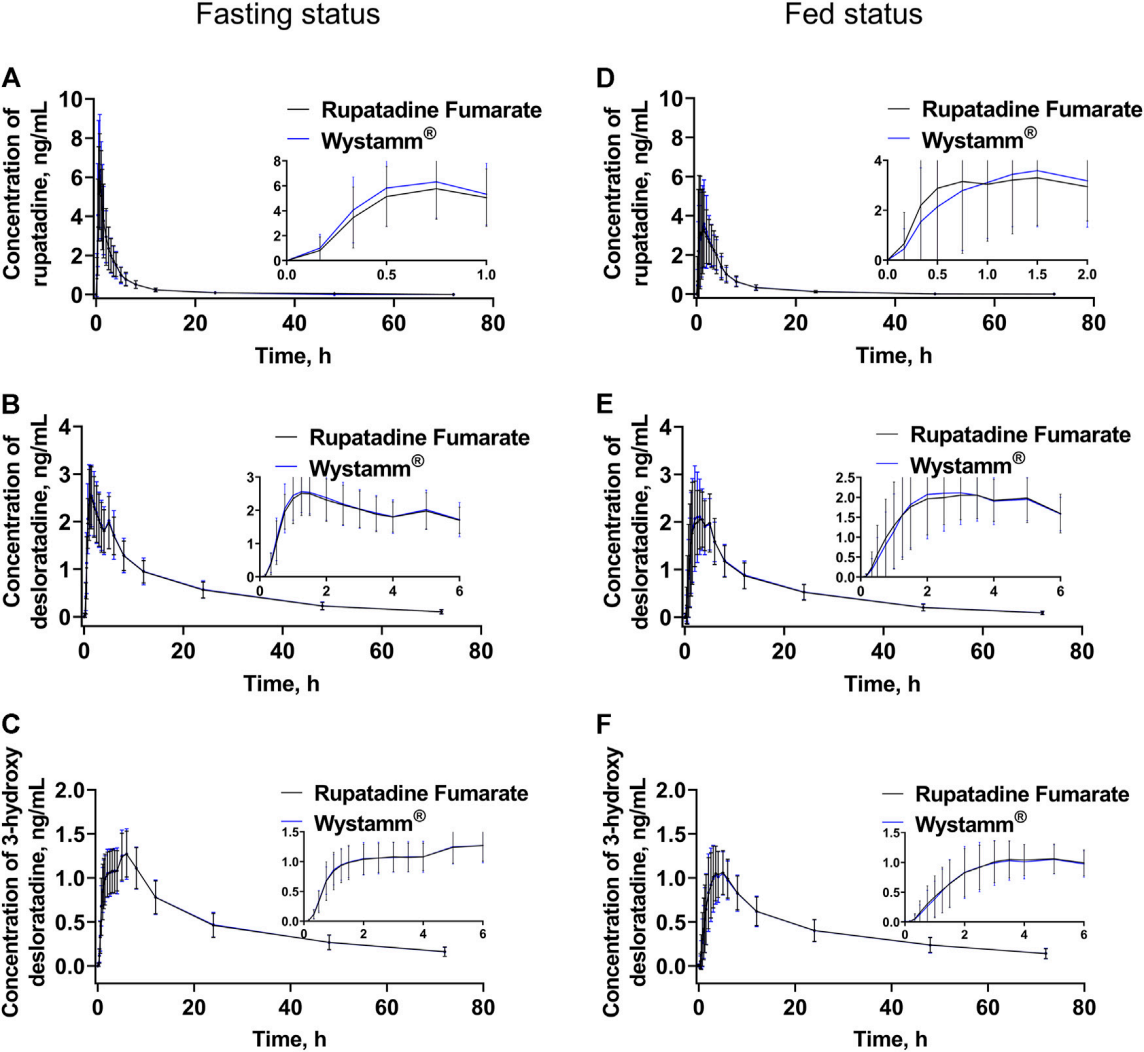
Plasma concentration–time curves. The mean (±SD) plasma concentration–time curves of rupatadine **(A)**, desloratadine **(B)**, and 3-hydroxydesloratadine **(C)** after a single oral administration of 10 mg rupatadine fumarate or Wystamm^®^ under the fasting status. The mean (±SD) plasma concentration–time curves of rupatadine **(D)**, desloratadine **(E)**, and 3-hydroxydesloratadine **(F)** after a single oral administration of 10 mg rupatadine fumarate or Wystamm^®^ under the postprandial status. Abbreviations: SD, standard deviation.

The PK parameters for the fasting and postprandial conditions are summarized in Table 2. The mean ± standard deviation (SD) plasma concentration–time curves of rupatadine (T vs. R) (Figures 2A, B) and those of desloratadine and 3-hydroxydesloratadine (Figure 2C–F) were drawn after four periods of administration under fasting and fed conditions.

**TABLE 2:** Pharmacokinetic parameters (mean ± SD) after the administration of rupatadine fumarate and Wystamm^®^ under fasting and fed conditions.

PK parameter	Fasting status		Fed status	
	Rupatadine fumarate (N = 36)	Wystamm^®^ (N = 36)	Rupatadine fumarate (N = 35)	Wystamm^®^ (N = 36)
Rupatadine				
AUC_0-∞_, h*ng/mL	19.57 ± 7.36 (37.63)	20.03 ± 8.05 (40.20)	20.98 ± 9.18 (43.77)	21.01 ± 7.83 (37.25)
AUC_0-t_, h*ng/mL	18.75 ± 7.12 (37.96)	19.20 ± 7.82 (40.74)	19.90 ± 8.99 (45.19)	19.90 ± 7.67 (38.53)
C_max_, ng/mL	6.85 ± 2.47 (35.98)	7.27 ± 2.97 (40.87)	5.05 ± 2.82 (55.79)	4.85 ± 2.42 (50.00)
T_max_, h	0.75 (0.33, 1.50)	0.75 (0.33, 1.50)	1.50 (0.32, 4.03)	1.26 (0.33, 5.00)
λ_z_, 1/h	0.1187 ± 0.0380 (32.0472)	0.1167 ± 0.0365 (31.2646)	0.1044 ± 0.0414 (39.6426)	0.0951 ± 0.0339 (35.6108)
t_1/2_, h	6.59 ± 2.90 (43.97)	6.60 ± 2.52 (38.15)	7.66 ± 3.08 (40.25)	8.20 ± 2.95 (36.05)
Desloratadine				
AUC_0-∞_, h*ng/mL	43.00 ± 11.11 (25.84)	43.65 ± 12.83 (29.39)	38.52 ± 10.70 (27.79)	39.02 ± 11.43 (29.30)
AUC_0-t_, h*ng/mL	40.11 ± 9.98 (24.87)	40.68 ± 11.40 (28.01)	36.20 ± 9.82 (27.13)	36.62 ± 10.50 (28.66)
C_max_, ng/mL	2.79 ± 0.74 (26.40)	2.83 ± 0.67 (23.77)	2.61 ± 0.78 (29.75)	2.64 ± 0.87 (33.18)
T_max_, h	1.25 (0.75, 5.05)	1.25 (0.75, 5.00)	2.76 (0.75, 6.00)	2.50 (1.25, 6.00)
λ_z_, 1/h	0.0369 ± 0.0041 (11.1009)	0.0368 ± 0.0039 (10.5225)	0.0386 ± 0.0046 (11.9597)	0.0385 ± 0.0050 (13.0129)
t_1/2_, h	19.03 ± 2.14 (11.23)	19.05 ± 2.27 (11.92)	18.23 ± 2.28 (12.50)	18.27 ± 2.26 (12.36)
3-Hydroxydesloratadine				
AUC_0-∞_, h*ng/mL	40.37 ± 10.13 (25.09)	40.27 ± 10.36 (25.73)	34.08 ± 10.60 (31.12)	33.95 ± 11.18 (32.95)
AUC_0-t_, h*ng/mL	32.86 ± 7.79 (23.71)	32.94 ± 8.10 (24.61)	27.52 ± 7.43 (27.00)	27.33 ± 7.70 (28.17)
C_max_, ng/mL	1.33 ± 0.29 (21.77)	1.32 ± 0.30 (22.43)	1.18 ± 0.29 (24.80)	1.17 ± 0.32 (27.36)
T_max_, h	5.04 (1.25, 8.00)	6.00 (1.00, 8.01)	3.50 (1.00, 8.02)	4.00 (1.50, 8.00)
λ_z_, 1/h	0.0223 ± 0.0032 (14.3205)	0.0226 ± 0.0027 (12.0043)	0.0225 ± 0.0029 (12.9735)	0.0227 ± 0.0032 (14.2940)
t_1/2_, h	31.68 ± 4.32 (13.65)	31.14 ± 3.89 (12.49)	31.37 ± 4.33 (13.80)	31.23 ± 4.59 (14.69)

Note: T_max_, median (min and max).

In the fasting condition, the mean ± SD (CV%) of the AUC_0-t_ values for T and R were 18.75 ± 7.12 h*ng/mL (37.96%) and 19.20 ± 7.82 h*ng/mL (40.74%), respectively; the AUC_0-∞_ values were 19.57 ± 7.36 h*ng/mL (37.63%) and 20.03 ± 8.05 h*ng/mL (40.20%), respectively; and the C_max_ values were 6.85 ± 2.47 ng/mL (35.98%) and 7.27 ± 2.97 ng/mL (40.87%), respectively. The median T_max_ was 0.75 for both medications. The mean ± SD (CV%) of the λ_z_ values for T and R were 0.1187 ± 0.0380 1/h (32.0472%) and 0.1167 ± 0.0365 1/h (31.2646%), respectively, and the t_1/2_ values were 6.59 ± 2.90 h (43.97%) and 6.60 ± 2.52 h (38.15%), respectively. The period, sequence, and formulation factors may affect the equivalence of the T formulation and R formulation in the bioequivalence study. The ANOVA results showed that a significant period effect was observed in the C_max_ (*p* = 0.0307), AUC_0-t_ (*p* = 0.0226), and AUC_0-∞_ (*p* = 0.0255) values of rupatadine. However, the bioequivalence of statistical differences in the administration period could still be recognized; there was no significant difference in C_max_, AUC_0-t_, and AUC_0-∞_ between the drug formulations and drug sequences (*p* > 0.05) (Table 3).

**TABLE 3:** Results of variance analysis of the main PK parameters after logarithmic transformation.

Fasting status (*p*-value)									
Effect factor	Rupatadine			Desloratadine			3-Hydroxydesloratadine		
	Cmax	AUC0-t	AUC_0-∞_	Cmax	AUC0-t	AUC_0-∞_	Cmax	AUC0-t	AUC_0-∞_
Sequence	0.0307^*^	0.0226^*^	0.0255^*^	0.3556	0.0561	0.0715	0.7227	0.0015^*^	0.0060^*^
Formulation	0.0821	0.1668	0.1665	0.2170	0.1035	0.1228	0.7276	0.9540	0.8662
Period	0.3425	0.6836	0.6665	0.3711	0.5775	0.6027	0.0333^*^	0.3220	0.3149

Fed status (*p*-value)									
Effect factor	Rupatadine			Desloratadine			3-Hydroxydesloratadine		
	Cmax	AUC0-t	AUC_0-∞_	Cmax	AUC0-t	AUC_0-∞_	Cmax	AUC0-t	AUC_0-∞_
Sequence	0.0626	0.2162	0.1796	0.0291^*^	0.3738	0.3286	0.1004	0.7965	0.4962
Formulation	0.2170	0.1262	0.1295	0.0471^*^	0.1205	0.1463	0.2577	0.0792	0.0772
Period	0.8427	0.6517	0.5969	0.8980	0.7654	0.7368	0.9000	0.4239	0.2431

Notes: *p* < 0.05 was considered statistically significant.

In the fed condition, C_max_, AUC_0-t_, and AUC_0-∞_ of subjects in the four periods were included in the PK parameter set of rupatadine, in addition to the parameters for the first period of the subject in group D who withdrew early from the study. The mean ± SD (CV%) of the AUC_0-t_ values for T and R were 19.90 ± 8.99 h*ng/mL (45.19%) and 19.90 ± 7.67 h*ng/mL (38.53%), respectively; the AUC_0-∞_ values were 20.98 ± 9.18 h*ng/mL (43.77%) and 21.01 ± 7.83 h*ng/mL (37.25%), respectively; and the C_max_ values were 5.05 ± 2.82 ng/mL (55.79%) and 4.85 ± 2.42 ng/mL (50.00%), respectively. The median T_max_ values for T and R were 1.50 and 1.26 h, respectively. The mean ± SD (CV%) of the λ_z_ values for T and R were 0.1044 ± 0.0414 1/h (39.6426%) and 0.0951 ± 0.0339 1/h (35.6108%), respectively, and the t_1/2_ values were 7.66 ± 3.08 h (40.25%) and 8.20 ± 2.95 h (36.05%), respectively.

### 3.3 Bioequivalence evaluation

The results of the bioequivalence evaluation between T and R under the fasting status and fed status are shown in Table 4.

**TABLE 4:** Bioequivalence statistics for PK parameters of rupatadine fumarate and Wystamm^®^ under the fasting and fed status.

PK parameter	Fasting status			Fed status		
	C_max_, ng/mL	AUC_0-t_, h*ng/mL	AUC_0-∞_, h*ng/mL	C_max_, ng/mL	AUC_0-t_, h*ng/mL	AUC_0-∞_, h*ng/mL
Rupatadine						
CV, %	33.66	19.07	18.85	28.79	13.36	12.97
Upper 95% Cl	−0.06	N/A	N/A	N/A	N/A	N/A
Evaluation method	RSABE	ABE	ABE	ABE	ABE	ABE
GMR (T/R), %	95.91	98.76	98.71	101.19	98.80	98.63
90% Cl, %	89.18–103.14	93.88–103.90	93.93–103.75	91.64–111.74	94.47–103.33	94.42–103.03
Power	93.76	100	100	99.02	100	100
Desloratadine						
CV, %	14.56	10.25	10.48	20.73	9.97	10.21
Evaluation method	ABE	ABE	ABE	ABE	ABE	ABE
GMR (T/R), %	97.95	98.97	99.01	99.58	99.50	99.43
90% Cl, %	94.28–101.77	95.96–102.07	95.91–102.20	94.31–105.14	96.78–102.30	99.13–103.56
Power	100	100	100	100	100	100
3-Hydroxydesloratadine						
CV, %	8.79	5.53	5.00	11.87	5.18	5.8
Evaluation method	ABE	ABE	ABE	ABE	ABE	ABE
GMR (T/R), %	104.47	101.18	101.32	99.72	100.77	101.20
90% Cl, %	101.02–108.03	99.19–103.21	99.13–103.56	99.15–103.44	99.18–102.40	99.50–102.94
Power	100	100	100	100	100	100

Notes: T, test; rupatadine fumarate; R, reference Wystamm^®^.

In the fasting status, 36 subjects completed the four-period study, and all PK parameters were calculated. Since the CV_WR_ value for C_max_ was >30%, the RSABE method was employed for equivalence evaluation. The T/R GMR (power) for C_max_ was 95.91% (93.76%), falling within the predefined interval of 80%–125%. Of note, the upper limit of the one-sided 95% CI was −0.06 < 0, indicating that the test drug rupatadine fumarate was bioequivalent to the reference drug Wystamm^®^ based on the endpoint C_max_. Furthermore, since the CV_WRs_ values for AUC_0-t_ and AUC_0-∞_ were below the threshold value (i.e., <30%), the ABE method was employed to evaluate the PK bioequivalence. The 90% CIs (power) of GMR were 93.88%–103.90% (100%) and 93.93%–103.75% (100%), respectively, both falling within the predefined interval (80%–125%) and, therefore, meeting the bioequivalence criteria for ABE.

In the fed condition, one subject dropped out after receiving the first single dose of the R formulation. Thus, the PK parameters of the T formulation were calculated in 35 subjects. Since the CV_WRs_ values for C_max_, AUC_0-t_, and AUC_0-∞_ were <30%, the bioequivalence evaluation was conducted using the ABE method. The 90% CIs (power) of the GMR for these PK parameters were 91.64%–111.74% (99.02%), 94.47%–103.33% (100%), and 94.47%–103.33% (100%), respectively, all falling within the predefined interval of 80%–125%; the upper limit of the two-sided 95% CI was <0. These results indicate that the test drug rupatadine fumarate was bioequivalent to the reference drug Wystamm^®^.

**FIGURE 3:**
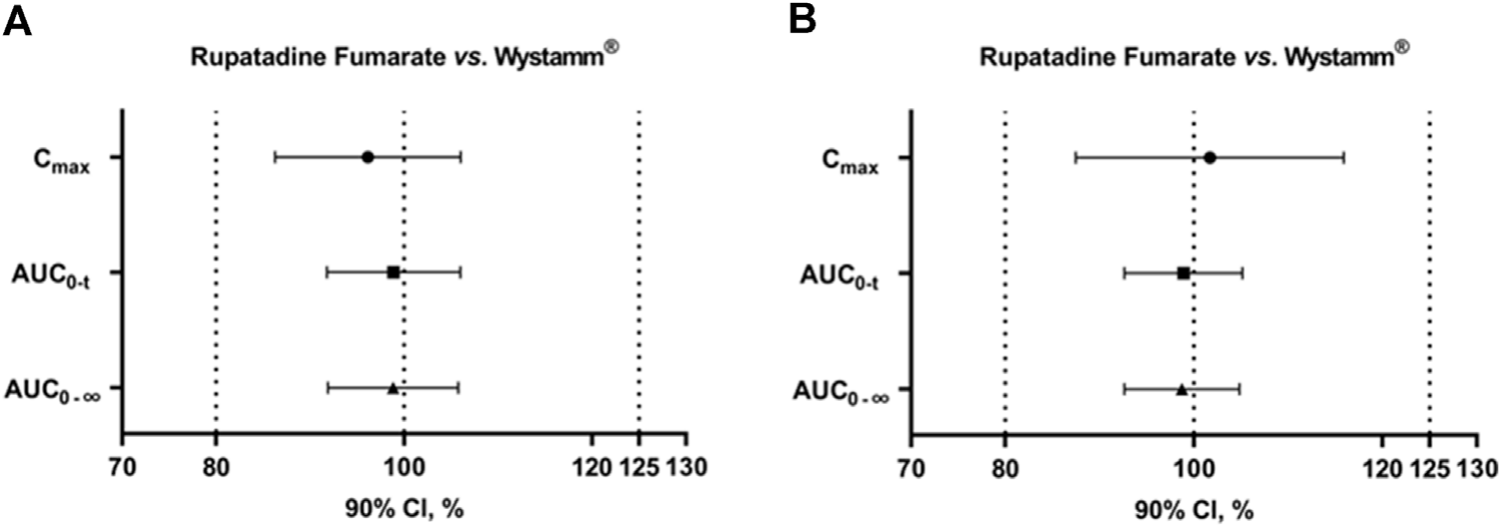
90% CIs of AUC_0-t_, C_max_, and AUC_0-∞_ for rupatadine fumarate and Wystamm^®^ under the fasting status **(A)** and postprandial status **(B)**. Abbreviations: AUC_0-t_, area under the rupatadine concentration–time curve from time 0 to the last detectable concentration; AUC_0-∞_, area under the rupatadine concentration–time curve from time 0 to infinity; CI, confidence interval; and C_max_, maximum observed rupatadine concentration.

According to the guidelines, the GMR for PK parameter endpoints of the major metabolites was not used as a basis to determine the bioequivalence of T and R formulations; notably, all values were within the predefined interval of 80%–125%. In summary, T and R were bioequivalent under both the fasting and fed conditions (Figure 3).

### 3.4 Safety evaluation

A summary of AEs by the systematic organ classification and preferred terminology for T and R under the fasting and postprandial conditions is presented in Table 5. Overall, both drugs exhibited a good safety profile in healthy Chinese subjects.

**TABLE 5:** Summary of AEs under fasting and fed condition.

Parameter		Fasting status		Fed status	
SOC	PT	Rupatadine fumarate (N = 36) N (%) [n]	Wystamm^®^ (N = 36) N (%) [n]	Rupatadine Fumarate (N = 35) N (%) [n]	Wystamm^®^ (N = 36) N (%) [n]
AEs		8 (22.2) [9]	9 (25.0) [12]	4 (11.4) [4]	4 (11.1) [8]
Investigations	Positive bacterial test	1 (2.8) [1]	1 (2.8) [1]	0 [0]	1 (2.8) [1]
	Reduced hemoglobin	2 (5.6) [2]	0 [0]	0 [0]	1 (2.8) [1]
	Elevated alanine aminotransferase	0 [0]	1 (2.8) [1]	0 [0]	0 [0]
	Elevated aspartate aminotransferase	0 [0]	1 (2.8) [1]	0 [0]	0 [0]
	Elevated low-density lipoprotein	0 [0]	0 [0]	0 [0]	1 (2.8) [1]
	Positive urine leukocyte	0 [0]	0 [0]	0 [0]	1 (2.8) [1]
	Elevated blood pressure	0 [0]	0 [0]	1 (2.9) [1]	0 [0]
	Elevated creatine phosphokinase	0 [0]	0 [0]	1 (2.9) [1]	0 [0]
Gastrointestinal disorders	Abdominal pain	1 (2.8) [1]	2 (5.6) [2]	0 [0]	1 (2.8) [1]
	Nausea	0 [0]	1 (2.8) [1]	0 [0]	0 [0]
	Toothache	1 (2.8) [1]	0 [0]	0 [0]	0 [0]
	Gastroesophageal reflux	1 (2.8) [1]	0 [0]	0 [0]	0 [0]
	Diarrhea	1 (2.8) [1]	0 [0]	0 [0]	1 (2.8) [1]
Blood and lymphatic disorder	Anemia	1 (2.8) [1]	2 (5.6) [2]	0 [0]	0 [0]
Neurological disorders	Dizziness	0 [0]	1 (2.8) [1]	0 [0]	0 [0]
	Headaches	0 [0]	1 (2.8) [1]	0 [0]	0 [0]
Infectious and infectious disorder	Upper respiratory tract infection	0 [0]	2 (5.6) [2]	0 [0]	0 [0]
	Urinary tract infection	0 [0]	0 [0]	1 (2.9) [1]	0 [0]
Metabolic and nutritional disorder	Hyperuricemia	1 (2.8) [1]	0 [0]	0 [0]	0 [0]
Cardiac disorders	Intraventricular conduction block	0 [0]	0 [0]	0 [0]	1 (2.8) [1]
	Ventricular extrasystole	0 [0]	0 [0]	1 (2.9) [1]	0 [0]
Eye disorder	Amaurosis	0 [0]	0 [0]	0 [0]	1 (2.8) [1]
Drug-related AEs		7 (19.4) 7	6 (16.7) 7	3 (8.6) 4	4 (11.1) [5]
Investigations	Positive bacterial test	1 (2.8) [1]	1 (2.8) [1]	0 [0]	1 (2.8) [1]
	Reduced hemoglobin	2 (5.6) [2]	0 [0]	0 [0]	0 [0]
	Elevated low-density lipoprotein	0 [0]	0 [0]	0 [0]	1 (2.8) [1]
	Elevated blood pressure	0 [0]	0 [0]	1 (2.9) [1]	0 [0]
	Elevated creatine phosphokinase	0 [0]	0 [0]	1 (2.9) [1]	0 [0]
Gastrointestinal disorders	Abdominal pain	1 (2.8) [1]	2 (5.6) [2]	0 [0]	1 (2.8) [1]
	Nausea	0 [0]	1 (2.8) [1]	0 [0]	0 [0]
	Diarrhea	1 (2.8) [1]	0 [0]	0 [0]	1 (2.8) [1]
Blood and lymphatic disorder	Anemia	1 (2.8) [1]	2 (5.6) [2]	0 [0]	0 [0]
Neurological disorders	Dizziness	0 [0]	1 (2.8) [1]	0 [0]	0 [0]
Infectious and infectious disorder	Urinary tract infection	0 [0]	0 [0]	1 (2.9) [1]	0 [0]
Metabolic and nutritional disorder	Hyperuricemia	1 (2.8) [1]	0 [0]	0 [0]	0 [0]
Cardiac disorders	Intraventricular conduction block	0 [0]	0 [0]	0 [0]	1 (2.8) [1]
	Ventricular extrasystole	0 [0]	0 [0]	1 (2.9) [1]	0 [0]

Notes: SOC, systematic organ classification; PT, preferred terminology; N, number of subjects with adverse events; AEs, adverse events; n, number of adverse events.

In the fasting status, seven (19.4%) and six (16.7%) subjects who received the T and R formulation, respectively, experienced drug-related treatment-emergent AEs (TEAEs). In the postprandial status, three (8.6%) and four (11.1%) subjects who received the T and R formulation, respectively, experienced TEAEs. The incidence of TEAEs was similar, and the severity of all AEs was grade 1; in addition, there were no severe AEs and AEs leading to subject withdrawal or death. Frequent TEAEs between the T and R groups were a positive bacterial test (2.8% vs. 2.8%, respectively), reduced hemoglobin (5.6% vs. 0.0%, respectively), abdominal pain (2.8% vs. 5.6%, respectively), anemia (2.8% vs. 5.6%, respectively), and upper respiratory tract infection (0.0% vs. 5.6%, respectively).

## 4 Discussion

Allergic rhinitis and CIU are chronic disorders that are associated with increased morbidity and thus have a major impact on the quality of life. Therefore, the control of the progression of anaphylactic disease is clinically significant. Oral antihistamines are the major pharmacological treatment. Rupatadine is a second-generation H1-receptor antagonist antihistamine and has potent PAF antagonist activity, the efficacy and safety of which have been demonstrated in clinical trials and clinical applications. However, the high cost of innovative products imposes a financial burden on patients. Thus, the exploitation of generic rupatadine extends the range of oral agents available for the treatment of allergic disorders.

The present trial was a phase Ⅰ, single-dose, randomized, open-label, four-period, crossover study on healthy Chinese subjects. It was designed to evaluate the bioequivalence and safety between the test drug rupatadine fumarate and the reference drug Wystamm^®^. The study involved 36 subjects under the fasting status and another 36 subjects under the fed status. One subject withdrew after the first administration under the fed status, and the remaining 71 subjects received the investigational drug (i.e., rupatadine fumarate or Wystamm^®^). The results revealed that the PK parameters (i.e., C_max_, AUC_0-t_, AUC_0–∞_, Tmax, t_1/2_, and λ_z_) were similar between T and R. PK parameters were also similar for desloratadine and 3-hydroxydesloratadine. All 90% CIs for the GMR of C_max_, AUC_0-t_, and AUC_0-∞_ were within the standard prespecified range (i.e., 80%–125%), indicating that T and R were bioequivalent.

The PK profiles of rupatadine in healthy subjects after a single dose of 10 mg under the fasting status have been well-established (Solans et al., 2008; Mullol et al., 2015; Sun et al., 2015). Rupatadine is rapidly absorbed within 45 min to 1 h after oral administration in adults, with a C_max_ of 2.3 ng/mL (Mullol et al., 2015). PK parameters, including C_max_, AUC_0-t_, and AUC_0-∞_, were consistent with those reported in previous studies, in which healthy Chinese subjects received rupatadine fumarate tablets (Zhuhai Kinhoo Pharmaceutical Co., Ltd., Zhuhai, China) (Sun et al., 2015). The C_max_, T_max_, and t_1/2_ values of two rupatadine formulations in our study were generally consistent with those reported by Solans et al. (2008), demonstrating rapid absorption and elimination. However, the AUC (AUC_0-t_ and AUC_0-∞_) in the present study was noticeably higher, indicating higher bioavailability and exposure level. Ethnic differences may be one of the factors responsible for this discrepancy. The majority of participants in the comparative study were European, whereas all subjects in our study were Chinese. The smaller body size and lower levels of cytochrome P450, family 3, and subfamily A (CYP3A) enzymes in Chinese compared with European individuals (two inter-ethnic physiological characteristics) mean a higher exposure to rupatadine (Ramamoorthy et al., 2015). In addition, the AUC_0-∞_ and t_1/2_ values of rupatadine were lower than those of desloratadine and 3-hydroxydesloratadine, indicating that both active metabolites may exert a lasting effect.

Consistent with a previous study, in this investigation, the effect of food on the PK parameters of rupatadine was mainly reflected in t_1/2_ and T_max_, which were prolonged in both the T and R groups (Solans et al., 2007). In fact, the PK profiles of rupatadine shifted to the right under the postprandial status; thus, it is highly probable that gastric emptying is responsible for the increase in t_1/2_ and T_max_. For both T and R, food consumption increased the AUC but decreased the C_max_ of rupatadine, while the C_max_ and AUC of desloratadine and 3-hydroxydesloratadine were decreased. This evidence indicated that food could accelerate the rate of rupatadine absorption, as reflected by an increase in AUC; nevertheless, it could slow it down, as reflected by a delay in T_max_ and a decrease in C_max_, which affected the formation of metabolites desloratadine and 3-hydroxydesloratadine. However, our primary objective was to estimate bioequivalence rather than the food effect; hence, the effect of food on the PK profile was imprecise in these two cohorts. In conclusion, despite the discrepancies in C_max_ and AUC compared with previous data, these differences did not affect the bioequivalence between T and R.

AEs were evaluated for both treatment groups in two separate cohorts; all AEs were TEAEs and rated as mild. The TEAEs included ventricular extrasystole, anemia, dizziness, headache, abdominal pain, positive bacterial tests, presence of albumin and red blood cells in urine, and elevated levels of creatine phosphokinase, alanine aminotransferase, and aspartate aminotransferase in blood, all of which have a low incidence rate. Headache was the most commonly reported AE in this study, with one and three cases occurring under the fasting status and fed status, respectively. However, a low rate of narcolepsy, which was frequently reported in European, Korean, Japanese, and Brazilian populations, was recorded in this study (Gimenez-Arnau et al., 2007; Mion et al., 2009; Hide, et al., 2019b; Won et al., 2021). Furthermore, there was no difference in the incidence of TEAEs between the T and R groups (*p* > 0.05). Therefore, we concluded that the test drug rupatadine fumarate and reference drug Wystamm^®^ have an equivalent safety profile.

Several limitations are present in this study. The recommended dose of two investigational drugs (10 mg bid) was used in this clinical trial, and dose titration was not selected. Further studies are warranted to verify the dose relationship between the investigational drugs and AEs. In addition, in this trial, the sample size was small, and physiological differences were limited. Although the requirements of a bioequivalence trial were met, this investigation is unable to provide a comprehensive evaluation of the PK profiles and safety of those drugs. Lastly, adolescents and children were not recruited in the trial; thus, the safety and effectiveness of the drugs in these populations should be further evaluated.

## 5 Conclusion

The results of the study confirmed that rupatadine fumarate 10 mg was bioequivalent to the reference drug Wystamm^®^ in healthy Chinese subjects under the fasting status and postprandial status. The two investigational drugs were generally well-tolerated and safe. These findings based on C_max_, AUC_0-t_, and AUC_0-∞_ in this clinical trial indicated that the test drug rupatadine fumarate could be an alternative to the reference drug Wystamm^®^ in China, thereby improving accessibility and reducing drug-related costs.

## Data Availability

The original contributions presented in the study are included in the article/Supplementary Material; further inquiries can be directed to the corresponding authors.
