# Supplementary material for: A single-dose, randomized, open-label, four-period, crossover equivalence trial comparing the clinical similarity of the proposed biosimilar rupatadine fumarate to reference Wystamm® in healthy Chinese subjects
Source: Front Pharmacol. 2024 May 17;15:1328142. doi: 10.3389/fphar.2024.1328142 (PMC11140027; doi:10.3389/fphar.2024.1328142)
Supplement: Supplementary file 1 [file Table1.docx]

Supplementary Table S1: PK parameters of rupatadine under the fasting status

| Parameters | pre dose | 10min | 20min | 0.5h | 0.75h | 1.0h | 1.25h | 1.5h | 2.0h | 2.5h | 3.0h | 3.5h | 4.0 h | 5.0h | 6.0h | 8.0h | 12.0h | 24.0h | 48.0h | 72.0h |
| --- | --- | --- | --- | --- | --- | --- | --- | --- | --- | --- | --- | --- | --- | --- | --- | --- | --- | --- | --- | --- |
|  | Rupatadine Fumarate | | | | | | | | | | | | | | | | | | | |
| Number | 72 | 72 | 72 | 72 | 72 | 72 | 72 | 72 | 72 | 72 | 72 | 72 | 72 | 72 | 72 | 72 | 72 | 72 | 72 | 72 |
| Mean of concentration | 0 | 0.812 | 3.45 | 5. 14 | 5.77 | 5.05 | 4.35 | 3.76 | 2.95 | 2.36 | 2 | 1.72 | 1.53 | 1.01 | 0.786 | 0.516 | 0.24 | 0.0924 | 0.0053 | 0 |
| SD | 0 | 1.08 | 2.45 | 2.41 | 2.45 | 2.31 | 2.06 | 1.88 | 1.46 | 1.09 | 0.867 | 0.755 | 0.616 | 0.383 | 0.348 | 0.2 | 0.0944 | 0.0449 | 0.0183 | 0 |
| CV% | - | 133 | 70.9 | 46.8 | 42.6 | 45.7 | 47.3 | 49.9 | 49.5 | 46.2 | 43.3 | 43.8 | 40.2 | 38 | 44.3 | 38.7 | 39.3 | 48.6 | 347 | - |
| Max | 0 | 4.88 | 11 | 11.7 | 17.9 | 18.2 | 15.2 | 12.9 | 9.95 | 7.54 | 5.94 | 4.82 | 3.68 | 2.31 | 2.48 | 1.24 | 0.678 | 0.294 | 0. 101 | 0 |
| Min | 0 | 0 | 0.0846 | 0.512 | 1.44 | 1.86 | 2.08 | 1.72 | 1.38 | 1. 10 | 0.868 | 0.814 | 0.656 | 0.492 | 0.306 | 0.237 | 0.0862 | 0 | 0 | 0 |
| Parameters | Wystamm® | | | | | | | | | | | | | | | | | | | |
| Number | 72 | 72 | 72 | 72 | 72 | 72 | 72 | 72 | 72 | 72 | 72 | 72 | 72 | 72 | 72 | 72 | 72 | 72 | 72 | 72 |
| Mean of concentration | 0 | 1 | 4.06 | 5.82 | 6.32 | 5.33 | 4.47 | 3.78 | 2.96 | 2.36 | 1.99 | 1.69 | 1.51 | 0.991 | 0.77 | 0.514 | 0.237 | 0.0947 | 0.0054 | 0 |
| SD | 0 | 1. 10 | 2.64 | 3.08 | 2.9 | 2.48 | 2. 18 | 1.75 | 1.33 | 1.08 | 0.902 | 0.785 | 0.7 | 0.429 | 0.303 | 0.204 | 0.0883 | 0.0468 | 0.0181 | 0 |
| CV% | - | 110 | 65 | 52.9 | 45.8 | 46.4 | 48.7 | 46. 1 | 44.9 | 45.7 | 45.2 | 46.5 | 46.4 | 43.3 | 39.4 | 39.7 | 37.4 | 49.5 | 338 | - |
| Max | 0 | 5.99 | 11.2 | 17.6 | 18.7 | 15.7 | 13.8 | 10 | 7.98 | 6.52 | 5.61 | 5. 11 | 4.53 | 2.77 | 1.73 | 1.2 | 0.56 | 0.28 | 0.0816 | 0 |
| Min | 0 | 0 | 0.0742 | 0.467 | 1.78 | 2.32 | 1.99 | 1.48 | 1. 13 | 0.935 | 0.807 | 0.791 | 0.672 | 0.404 | 0.319 | 0. 168 | 0.0769 | 0 | 0 | 0 |

Supplementary Table S2: PK parameters of rupatadine under the fed status

| Parameters | pre dose | 10min | 20min | 0.5h | 0.75h | 1.0h | 1.25h | 1.5h | 2.0h | 2.5h | 3.0h | 3.5h | 4.0 h | 5.0h | 6.0h | 8.0h | 12.0h | 24.0h | 48.0h | 72.0h |
| --- | --- | --- | --- | --- | --- | --- | --- | --- | --- | --- | --- | --- | --- | --- | --- | --- | --- | --- | --- | --- |
|  | Rupatadine Fumarate | | | | | | | | | | | | | | | | | | | |
| Number | 70 | 70 | 69 | 70 | 70 | 70 | 70 | 70 | 70 | 70 | 70 | 70 | 70 | 70 | 70 | 70 | 70 | 70 | 70 | 70 |
| Mean of concentration | 0 | 0.658 | 2.2 | 2.88 | 3. 15 | 3.04 | 3.21 | 3.3 | 2.94 | 2.66 | 2.45 | 2.26 | 1.99 | 1.41 | 1.02 | 0.654 | 0.333 | 0. 128 | 0.0139 | 0.000899 |
| SD | 0 | 1.26 | 3. 15 | 3. 18 | 2.88 | 2.28 | 2. 16 | 1.91 | 1.39 | 1. 15 | 1.09 | 0.978 | 0.946 | 0.677 | 0.45 | 0.279 | 0. 150 | 0.0675 | 0.0332 | 0.00752 |
| CV% | - | 192 | 143 | 110 | 91.5 | 75 | 67.3 | 58 | 47.2 | 43.3 | 44.5 | 43.3 | 47.5 | 48. 1 | 44 | 42.8 | 44.9 | 52.6 | 238 | 837 |
| Max | 0 | 5.49 | 13.4 | 13.3 | 14.5 | 9.97 | 11.7 | 10.3 | 9.66 | 8.97 | 8.63 | 7. 19 | 6.58 | 4.2 | 2.83 | 1.89 | 1.02 | 0.441 | 0. 181 | 0.0629 |
| Min | 0 | 0 | 0 | 0 | 0 | 0.0814 | 0. 162 | 0.206 | 0.438 | 0.715 | 0.874 | 0.752 | 0.72 | 0.511 | 0.401 | 0.28 | 0. 134 | 0 | 0 | 0 |
| Parameters | Wystamm® | | | | | | | | | | | | | | | | | | | |
| Number | 71 | 71 | 71 | 71 | 71 | 70 | 71 | 71 | 71 | 71 | 71 | 71 | 71 | 71 | 71 | 71 | 71 | 71 | 71 | 71 |
| Mean of concentration | 0 | 0.45 | 1.54 | 2. 14 | 2.79 | 3. 11 | 3.44 | 3.59 | 3. 18 | 2.79 | 2.49 | 2.23 | 2.01 | 1.38 | 1.01 | 0.632 | 0.337 | 0. 129 | 0.0156 | 0.000763 |
| SD | 0 | 0.812 | 2. 16 | 2.38 | 2.4 | 2.24 | 2. 10 | 2.25 | 1.86 | 1.22 | 1.07 | 0.782 | 0.776 | 0.535 | 0.37 | 0.232 | 0. 130 | 0.0574 | 0.0304 | 0.00643 |
| CV% | - | 180 | 141 | 111 | 86. 1 | 71.8 | 61. 1 | 62.6 | 58.4 | 43.5 | 42.8 | 35 | 38.5 | 38.9 | 36.5 | 36.8 | 38.5 | 44.4 | 195 | 843 |
| Max | 0 | 3.85 | 10.8 | 10.7 | 14.9 | 11.5 | 9.97 | 11.2 | 12.5 | 7.06 | 6.71 | 4.37 | 4.97 | 3 | 1.98 | 1.22 | 0.719 | 0.289 | 0. 106 | 0.0542 |
| Min | 0 | 0 | 0 | 0 | 0 | 0 | 0 | 0 | 0 | 0 | 0.443 | 0.895 | 0.797 | 0.467 | 0.4 | 0.291 | 0. 161 | 0 | 0 | 0 |

Supplementary Table S3: PK parameters of desloratadine under the fasting status

| Parameters | pre dose | 10min | 20min | 0.5h | 0.75h | 1.0h | 1.25h | 1.5h | 2.0h | 2.5h | 3.0h | 3.5h | 4.0 h | 5.0h | 6.0h | 8.0h | 12.0h | 24.0h | 48.0h | 72.0h |
| --- | --- | --- | --- | --- | --- | --- | --- | --- | --- | --- | --- | --- | --- | --- | --- | --- | --- | --- | --- | --- |
|  | Rupatadine Fumarate | | | | | | | | | | | | | | | | | | | |
| Number | 0.000925 | 0.0279 | 0.384 | 1.03 | 1.96 | 2.35 | 2.51 | 2.5 | 2.31 | 2. 16 | 2.03 | 1.88 | 1.8 | 1.98 | 1.7 | 1.28 | 0.946 | 0.562 | 0.226 | 0. 103 |
| Mean of concentration | 0.00556 | 0.0441 | 0.327 | 0.654 | 0.855 | 0.75 | 0.662 | 0.652 | 0.637 | 0.595 | 0.549 | 0.525 | 0.449 | 0.548 | 0.394 | 0.312 | 0.237 | 0. 169 | 0.0742 | 0.0421 |
| SD | 601 | 158 | 85. 1 | 63.2 | 43.5 | 31.9 | 26.3 | 26. 1 | 27.5 | 27.6 | 27. 1 | 28 | 24.9 | 27.6 | 23. 1 | 24.4 | 25. 1 | 30. 1 | 32.8 | 40.9 |
| CV% | 0.0378 | 0. 195 | 1.56 | 3.75 | 4. 18 | 4.44 | 4.9 | 4.29 | 4.59 | 4.05 | 3.9 | 4.25 | 3.2 | 4.42 | 2.84 | 2. 17 | 1.7 | 1.09 | 0.403 | 0.216 |
| Max | 0 | 0 | 0 | 0.0804 | 0.318 | 0.673 | 1.24 | 1.22 | 1.32 | 1.3 | 1.22 | 1. 14 | 1.08 | 1.2 | 0.976 | 0.711 | 0.52 | 0.311 | 0. 118 | 0.0446 |
| Min | 0.000925 | 0.0279 | 0.384 | 1.03 | 1.96 | 2.35 | 2.51 | 2.5 | 2.31 | 2. 16 | 2.03 | 1.88 | 1.8 | 1.98 | 1.7 | 1.28 | 0.946 | 0.562 | 0.226 | 0. 103 |
| Parameters | Wystamm® | | | | | | | | | | | | | | | | | | | |
| Number | 72 | 72 | 72 | 72 | 72 | 72 | 72 | 72 | 72 | 72 | 72 | 72 | 72 | 72 | 72 | 72 | 72 | 72 | 72 | 72 |
| Mean of concentration | 0.00085 | 0.0354 | 0.423 | 1. 13 | 2.06 | 2.45 | 2.56 | 2.54 | 2.38 | 2. 19 | 2.04 | 1.92 | 1.81 | 2.02 | 1.72 | 1.29 | 0.954 | 0.573 | 0.228 | 0. 104 |
| SD | 0.00509 | 0.0438 | 0.298 | 0.641 | 0.735 | 0.744 | 0.628 | 0.648 | 0.676 | 0.648 | 0.582 | 0.526 | 0.504 | 0.586 | 0.514 | 0.364 | 0.281 | 0. 182 | 0.084 | 0.0473 |
| CV% | 599 | 124 | 70.5 | 56.7 | 35.7 | 30.3 | 24.6 | 25.5 | 28.4 | 29.6 | 28.5 | 27.3 | 27.8 | 29 | 29.9 | 28.2 | 29.4 | 31.8 | 36.8 | 45.4 |
| Max | 0.0337 | 0. 197 | 1.23 | 2.86 | 3.67 | 4.8 | 4.69 | 4.45 | 4.64 | 4.62 | 4.5 | 4.38 | 4.06 | 4.97 | 4.32 | 2.97 | 2. 10 | 1.28 | 0.518 | 0.275 |
| Min | 0 | 0 | 0 | 0.0561 | 0.384 | 0.794 | 0.935 | 1.22 | 0.991 | 1. 18 | 1.21 | 1. 15 | 1. 14 | 1.21 | 1.01 | 0.686 | 0.533 | 0.306 | 0. 115 | 0.046 |

Supplementary Table S4: PK parameters of desloratadine under the fed status

| Parameters | pre dose | 10min | 20min | 0.5h | 0.75h | 1.0h | 1.25h | 1.5h | 2.0h | 2.5h | 3.0h | 3.5h | 4.0 h | 5.0h | 6.0h | 8.0h | 12.0h | 24.0h | 48.0h | 72.0h |
| --- | --- | --- | --- | --- | --- | --- | --- | --- | --- | --- | --- | --- | --- | --- | --- | --- | --- | --- | --- | --- |
|  | Rupatadine Fumarate | | | | | | | | | | | | | | | | | | | |
| Number | 70 | 70 | 69 | 70 | 70 | 70 | 70 | 70 | 70 | 70 | 70 | 70 | 70 | 70 | 70 | 70 | 70 | 70 | 70 | 70 |
| Mean of concentration | 0 | 0.0253 | 0.245 | 0.569 | 0.975 | 1.29 | 1.55 | 1.76 | 1.96 | 1.99 | 2.05 | 2.05 | 1.92 | 1.98 | 1.59 | 1. 17 | 0.868 | 0.521 | 0.201 | 0.0852 |
| SD | 0 | 0.0581 | 0.384 | 0.723 | 0.974 | 1.09 | 1. 11 | 1.07 | 0.901 | 0.675 | 0.615 | 0.581 | 0.523 | 0.615 | 0.483 | 0.33 | 0.271 | 0. 163 | 0.0697 | 0.0355 |
| CV% | - | 230 | 157 | 127 | 99.8 | 85. 1 | 71.6 | 61 | 46 | 34 | 30 | 28.4 | 27.3 | 31 | 30.4 | 28.2 | 31.3 | 31.2 | 34.7 | 41.6 |
| Max | 0 | 0.313 | 1.8 | 3.66 | 4.76 | 4.76 | 4.74 | 4.96 | 4.57 | 3.91 | 3.71 | 4. 14 | 3.4 | 4.2 | 3. 16 | 2. 16 | 1.72 | 0.985 | 0.437 | 0. 192 |
| Min | 0 | 0 | 0 | 0 | 0 | 0 | 0.0368 | 0.0637 | 0. 174 | 0.227 | 0.536 | 0.983 | 1.03 | 0.989 | 0.872 | 0.676 | 0.519 | 0.289 | 0.0957 | 0.0357 |
| Parameters | Wystamm® | | | | | | | | | | | | | | | | | | | |
| Number | 71 | 71 | 71 | 71 | 71 | 71 | 71 | 71 | 71 | 71 | 71 | 71 | 71 | 71 | 71 | 71 | 71 | 71 | 71 | 71 |
| Mean of concentration | 0 | 0.0205 | 0. 189 | 0.431 | 0.811 | 1. 15 | 1.56 | 1.82 | 2.07 | 2. 10 | 2. 11 | 2.05 | 1.9 | 1.95 | 1.58 | 1. 19 | 0.885 | 0.527 | 0.204 | 0.0881 |
| SD | 0 | 0.0435 | 0.276 | 0.559 | 0.818 | 0.933 | 1.06 | 1.09 | 1. 11 | 0.949 | 0.794 | 0.648 | 0.586 | 0.539 | 0.417 | 0.338 | 0.294 | 0. 162 | 0.0754 | 0.0374 |
| CV% | - | 212 | 146 | 130 | 101 | 81. 1 | 68 | 59.8 | 53.4 | 45. 1 | 37.5 | 31.6 | 30.8 | 27.6 | 26.3 | 28.4 | 33.3 | 30.8 | 37. 1 | 42.4 |
| Max | 0 | 0.229 | 1. 13 | 2.84 | 3.91 | 3.97 | 4.62 | 4.8 | 5.89 | 5.45 | 5 | 3.9 | 3.45 | 3.29 | 2.54 | 2.23 | 1.93 | 0.992 | 0.444 | 0. 192 |
| Min | 0 | 0 | 0 | 0 | 0 | 0 | 0 | 0 | 0 | 0 | 0.0677 | 0.559 | 0.859 | 0.919 | 0.898 | 0.657 | 0.472 | 0.281 | 0.0978 | 0.0342 |

Supplementary Table S5: PK parameters of 3-hydroxy desloratadine under the fasting status

| Parameters | pre dose | 10min | 20min | 0.5h | 0.75h | 1.0h | 1.25h | 1.5h | 2.0h | 2.5h | 3.0h | 3.5h | 4.0 h | 5.0h | 6.0h | 8.0h | 12.0h | 24.0h | 48.0h | 72.0h |
| --- | --- | --- | --- | --- | --- | --- | --- | --- | --- | --- | --- | --- | --- | --- | --- | --- | --- | --- | --- | --- |
|  | Rupatadine Fumarate | | | | | | | | | | | | | | | | | | | |
| Number | 72 | 72 | 72 | 72 | 72 | 72 | 72 | 72 | 72 | 72 | 72 | 72 | 72 | 72 | 72 | 72 | 72 | 72 | 72 | 72 |
| Mean of concentration | 0.0072 | 0.0142 | 0. 112 | 0.324 | 0.672 | 0.841 | 0.936 | 0.982 | 1.04 | 1.06 | 1.08 | 1.07 | 1.08 | 1.24 | 1.27 | 1. 11 | 0.778 | 0.461 | 0.268 | 0. 162 |
| SD | 0.0145 | 0.0193 | 0.0797 | 0. 182 | 0.324 | 0.315 | 0.286 | 0.288 | 0.254 | 0.242 | 0.249 | 0.242 | 0.229 | 0.268 | 0.262 | 0.234 | 0. 188 | 0. 137 | 0.0834 | 0.0537 |
| CV% | 201 | 136 | 71.3 | 56.3 | 48.2 | 37.5 | 30.6 | 29.3 | 24.4 | 22.9 | 23 | 22.7 | 21. 1 | 21.5 | 20.6 | 21. 1 | 24.2 | 29.8 | 31. 1 | 33. 1 |
| Max | 0.0519 | 0.0732 | 0.324 | 0.746 | 2.02 | 2.22 | 1.97 | 2.34 | 1.83 | 1.78 | 1.79 | 1.97 | 1.71 | 2.06 | 2.01 | 1.83 | 1.27 | 0.842 | 0.503 | 0.364 |
| Min | 0 | 0 | 0 | 0 | 0. 147 | 0.318 | 0.398 | 0.418 | 0.517 | 0.56 | 0.574 | 0.644 | 0.635 | 0.705 | 0.87 | 0.712 | 0.416 | 0. 196 | 0. 141 | 0.0848 |
| Parameters | Wystamm® | | | | | | | | | | | | | | | | | | | |
| Number | 72 | 72 | 72 | 72 | 72 | 72 | 72 | 72 | 72 | 72 | 72 | 72 | 72 | 72 | 72 | 72 | 72 | 72 | 72 | 72 |
| Mean of concentration | 0.0087 | 0.0143 | 0. 119 | 0.341 | 0.682 | 0.863 | 0.946 | 0.991 | 1.05 | 1.06 | 1.07 | 1.08 | 1.08 | 1.25 | 1.27 | 1. 11 | 0.78 | 0.468 | 0.266 | 0. 161 |
| SD | 0.0152 | 0.0187 | 0.0769 | 0. 172 | 0.23 | 0.255 | 0.257 | 0.262 | 0.275 | 0.268 | 0.249 | 0.256 | 0.261 | 0.292 | 0.288 | 0.239 | 0. 196 | 0. 140 | 0.0842 | 0.0521 |
| CV% | 174 | 131 | 64.7 | 50.4 | 33.7 | 29.6 | 27.2 | 26.4 | 26.2 | 25.4 | 23.3 | 23.7 | 24. 1 | 23.5 | 22.6 | 21.6 | 25.2 | 29.9 | 31.6 | 32.3 |
| Max | 0.054 | 0.071 | 0.352 | 0.796 | 1. 11 | 1.39 | 1.68 | 1.89 | 1.85 | 1.8 | 1.65 | 1.66 | 1.73 | 2. 15 | 2. 14 | 1.76 | 1.24 | 0.926 | 0.579 | 0.353 |
| Min | 0 | 0 | 0 | 0 | 0. 110 | 0.308 | 0.489 | 0.465 | 0.537 | 0.562 | 0.609 | 0.543 | 0.556 | 0.731 | 0.755 | 0.631 | 0.446 | 0.257 | 0. 150 | 0.0789 |

Supplementary Table S6: PK parameters of 3-hydroxy desloratadine under the fed status

| Parameters | pre dose | 10min | 20min | 0.5h | 0.75h | 1.0h | 1.25h | 1.5h | 2.0h | 2.5h | 3.0h | 3.5h | 4.0 h | 5.0h | 6.0h | 8.0h | 12.0h | 24.0h | 48.0h | 72.0h |
| --- | --- | --- | --- | --- | --- | --- | --- | --- | --- | --- | --- | --- | --- | --- | --- | --- | --- | --- | --- | --- |
|  | Rupatadine Fumarate | | | | | | | | | | | | | | | | | | | |
| Number | 69 | 70 | 69 | 70 | 70 | 70 | 70 | 70 | 70 | 70 | 70 | 70 | 70 | 70 | 70 | 70 | 70 | 70 | 70 | 70 |
| Mean of concentration | 0.00402 | 0.0057 | 0.0534 | 0. 156 | 0.302 | 0.422 | 0.535 | 0.639 | 0.826 | 0.915 | 1.01 | 1.05 | 1.04 | 1.09 | 0.992 | 0.83 | 0.62 | 0.402 | 0.238 | 0. 140 |
| SD | 0.013 | 0.0132 | 0.0848 | 0. 199 | 0.303 | 0.351 | 0.383 | 0.399 | 0.405 | 0.371 | 0.35 | 0.312 | 0.261 | 0.25 | 0.22 | 0. 192 | 0. 163 | 0. 126 | 0.0841 | 0.0562 |
| CV% | 324 | 231 | 159 | 128 | 100 | 83.2 | 71.6 | 62.3 | 49 | 40.6 | 34.6 | 29.8 | 25. 1 | 22.9 | 22.2 | 23. 1 | 26.3 | 31.4 | 35.4 | 40 |
| Max | 0.0749 | 0.0588 | 0.403 | 1.06 | 1.48 | 1.49 | 1.46 | 1.48 | 1.78 | 1.84 | 2. 18 | 2. 14 | 1.87 | 1.94 | 1.5 | 1.24 | 1.05 | 0.743 | 0.496 | 0.362 |
| Min | 0 | 0 | 0 | 0 | 0 | 0 | 0 | 0 | 0.0482 | 0.0843 | 0. 172 | 0.328 | 0.516 | 0.608 | 0.563 | 0.43 | 0.335 | 0. 183 | 0. 115 | 0.0643 |
| Parameters | Wystamm® | | | | | | | | | | | | | | | | | | | |
| Number | 71 | 71 | 71 | 71 | 71 | 71 | 71 | 71 | 71 | 71 | 71 | 71 | 71 | 71 | 71 | 71 | 71 | 71 | 71 | 71 |
| Mean of concentration | 0.00641 | 0.00791 | 0.0459 | 0. 126 | 0.263 | 0.385 | 0.522 | 0.647 | 0.833 | 0.923 | 0.99 | 1.03 | 1.01 | 1.05 | 0.973 | 0.826 | 0.619 | 0.4 | 0.237 | 0. 141 |
| SD | 0.0161 | 0.0163 | 0.0672 | 0. 162 | 0.27 | 0.32 | 0.367 | 0.401 | 0.439 | 0.417 | 0.379 | 0.329 | 0.288 | 0.246 | 0.221 | 0.205 | 0. 174 | 0. 125 | 0.0872 | 0.0606 |
| CV% | 250 | 207 | 146 | 128 | 103 | 83. 1 | 70.2 | 62 | 52.7 | 45.2 | 38.3 | 32 | 28.4 | 23.4 | 22.7 | 24.8 | 28. 1 | 31. 1 | 36.8 | 43 |
| Max | 0.0689 | 0.0584 | 0.262 | 0.762 | 1.23 | 1.43 | 1.6 | 1.89 | 2.07 | 2.28 | 2.39 | 2. 11 | 2.07 | 1.59 | 1.43 | 1.44 | 1.09 | 0.802 | 0.551 | 0.361 |
| Min | 0 | 0 | 0 | 0 | 0 | 0 | 0 | 0 | 0 | 0 | 0.443 | 0.895 | 0.797 | 0.467 | 0.4 | 0.291 | 0. 161 | 0 | 0 | 0 |
